# Effects of Booking Horizon Reduction on Cancellation Rates: An Experimental Analysis in Pediatric Outpatient Care

**DOI:** 10.1177/23814683241298673

**Published:** 2024-11-18

**Authors:** Benjamin Ravenscroft, Hossein Abouee Mehrizi, Brendan Wylie-Toal

**Affiliations:** Department of Management Science and Engineering, University of Waterloo, Waterloo, ON, Canada; Department of Management Science and Engineering; Faculty of Engineering, University of Waterloo, Waterloo, ON, Canada; Director of Research and Innovation, KidsAbility, Kitchener, ON, Canada

**Keywords:** cancellation rates, lead time, scheduling, booking horizon, patient no-shows

## Abstract

**Background.** The time between booking an appointment and the appointment taking place, known as lead time, has been identified as a predictor of cancellation and no-show probability in health care settings. Understanding the impact of reducing permissible lead times, that is, the booking horizon, at a policy level in an outpatient care setting is important when mitigating costly cancellation and no-show rates. Few studies have researched this in an observational or experimental setting. **Methods.** We leveraged longitudinal observational data from an outpatient pediatric rehabilitation organization in Ontario, Canada, consisting of 73,482 visits between June 2021 and October 2023. This organization reduced its booking horizon at the policy level from 12 to 4 wk in February 2023. Using 2 interrupted time-series approaches, we estimated the change in level, slope, and variance of the weekly combined last-minute cancellation and no-show rate associated with the policy change. **Results.** It is estimated that reducing the booking horizon is associated with an absolute reduction in the weekly rate of last-minute cancellations and no-shows of 1.02% to 1.85% (a relative reduction of 8.07%–15.70%). Furthermore, the variance dropped by 48.18%. **Conclusion.** Reducing the appointment booking horizon is associated with a significant reduction in the rate and variance of costly last-minute cancellations and no-shows. The reduced variance can also help enable effective usage of strategies such as overbooking for organizations seeking further approaches to mitigating the negative effects of no-shows.

**Highlights:**

- This study uses interrupted time-series approaches to assess the effects of reducing the appointment booking horizon at a policy level on last-minute cancellations and no-shows in a pediatric outpatient care setting.
- Reducing the permissible booking horizon from up to 3 mo to up to 4 wk is associated with a significant reduction in the rate of last-minute cancellations and no-shows.
- The shortened booking horizon policy is associated with a significant drop in the variance of last-minute cancellations and no-show rates, which is valuable in settings where overbooking occurs.

## Introduction

Extensive wait times for pediatric rehabilitation services, particularly speech-language therapy, are a growing issue in Canada and other parts of the world.^1^ Demand has steadily outpaced clinical supply in recent years, an issue that was exacerbated by the COVID-19 pandemic and associated public health restrictions causing service reductions and closures. In Ontario, Canada, most speech-language pathology (SLP) treatment is provided by nonprofit children’s treatment centers (CTCs). These organizations have been struggling to keep up with service demand due in part to an inability to increase clinical capacity,^2^ which is a function of the number of working clinicians and their effective treatment capacity. This staffing shortage is not unique to Canada but rather endemic in the field of speech-language therapy.^3,4^ In Canada, even with increased budgets, organizations report a dearth of available clinicians to hire, which severely hampers their ability to expand service capacity.^2^ Thus, organizations such as our partner in southwestern Ontario have become increasingly interested in operational methods that can be leveraged to increase system throughput and subsequently decrease wait times.

Although absolute clinical capacity is difficult to increase without hiring more clinicians or extending working hours, system efficiency can be improved within the current constraints, increasing the flow of patients. Cancellations, particularly those within 24 h of the scheduled appointment time (i.e., last-minute cancellations), and no-shows are a significant source of inefficiency in health care systems. If other patients cannot fill these appointment slots, cancellations effectively reduce the number of patients being serviced compared with the total clinical capacity. The format of many services offered by CTCs creates barriers to effectively implementing common solutions used in other health care or service settings, such as open-access scheduling and overbooking.^5,6^ For example, patients are assigned to a single clinician and receive repeat treatment, ideally with a specific frequency intended to maximize clinical efficacy. This precludes using open-access scheduling, which refers to systems in which patients are provided an appointment within 24 h of calling the clinic, no matter the reason,^7^ as it interferes with the maintenance of appropriate appointment intervals. Furthermore, clinicians have a fixed, union-determined, limit on working hours per week. This makes overbooking difficult as it runs the risk of overbooked patients going untreated by clinicians when all regularly scheduled appointments on a day occur. In addition, in the case of early years treatment (patients <3 y of age), there are logistical constraints related to needing to provide (potentially long periods of) childcare for waiting overbooked patients.

Motivated by these challenges, we investigate avenues for decreasing the rate of cancellations and no-shows, rather than mitigating their impacts when they occur. The root causes of patient cancellations and no-shows vary. Regarding outpatient clinics, they may include adverse weather, unforeseen events in the patient’s schedule, forgetfulness, or unforeseen scheduling conflicts.^8^ Much of the existing literature focuses on studying predictors of no-shows versus shows, with studies citing longer lead times, prior attendance behavior, distance to the appointment site, and the number of previously scheduled appointments as significant predictors of cancellation likelihood.^9–13^

Comparatively few studies exist assessing the differences in the behavior of cancellations and no-shows.^14–17^ Furthermore, a limited number of studies distinguish between different types of cancellations, such as last-minute cancellations (i.e., those that occur within 24 h of the appointment time) and more advanced cancellations. Two studies identified a bimodal distribution of cancellation rates in relation to appointment lead time.^17,18^ They note a peak in cancellations soon after the appointment is booked and then again close to the scheduled appointment time. This is an important distinction, as appointment slots canceled well in advance have a higher likelihood of being filled with another patient compared with those canceled with short notice, which are more likely to incur staff idle time.

The existing literature often cites appointment lead times as being positively associated with appointment no-show likelihood^11,13^; however, some studies do not identify this association in their data.^19^ The limited studies that consider cancellations also identify a positive association between lead time and cancellation likelihood.^14,15,17,20^ Exact reasons explaining why longer lead times are often associated with an increased likelihood of cancellation or no-show are rarely provided in the literature. Briatore et al.^8^ administered questionnaires to outpatient clinic patients eliciting their reasons for missing an appointment. The most frequently reported causes were forgetting the appointment or the development of unexpected competing events.^8^ We posit that the likelihood of forgetting an appointment increases with lead time, caused by transience (i.e., the decline of memory performance over time),^21^ and thereby increases the probability of no-showing. Furthermore, we suggest the likelihood of unexpected competing events arising increases with lead time, as the uncertainty of an individual’s personal/work schedule increases further into the future they look ahead. Unexpected competing events could lead to no-shows or cancellations when individuals realize ahead of their scheduled appointments that they have a conflict and need to cancel/reschedule.

The relationship between lead time and cancellations/no-shows supports limiting the booking horizon (i.e., the maximum allowable lead time) to reduce cancellations and no-shows. It is important to note that a booking horizon policy can either have a hard or soft maximum on the allowable lead times. In the first case, if there are no empty appointment slots within the horizon, a patient’s request for service will be denied. This is the type of booking horizon the modeling literature typically considers.^17,22^ In the second case, if after a visit a clinician has no empty slots within the booking horizon, they cannot simply deny this patient their next treatment. The hope is that instead, the clinician will try to wait and follow up with the client later to book their next appointment within, or as close as possible to, the booking horizon maximum.

The relevant literature focuses on settings with hard maximums. For example, Liu.^22^ developed an M/M/1/K queuing model to determine an optimal booking horizon to minimize no-shows, and Leeftink et al.^17^ adapted this model to consider cancellations. The authors of these studies verified the impacts of these models using simulation studies. For instance, Leeftink et al.^17^ estimated that shortening the booking horizon to 6 d at one institution they examined would result in a 7.5% reduction in cancellation rates and a 2.5% reduction in no-show rates. However, to the best of our knowledge, no study has investigated the implementation of an optimal booking horizon policy, particularly with a soft horizon and a focus on cancellation as well as no-show effects, in a real clinical setting.

In this work, we explore the effects of reducing the booking horizon at a policy level on the combined last-minute cancellation and no-show rate at a large CTC in southwestern Ontario. The optimal horizon choice is informed by the queueing models put forth by Liu^22^ and Leeftink et al.^17^ Our main contributions validate that reducing the booking horizon is associated with a significant reduction in the rate of costly last-minute cancellations and no-shows, even in a setting without a hard maximum horizon.

The primary motivation for this work was to empirically measure the effect of shortening lead times on last-minute cancellation and no-show rates, which conversely increases resource utilization and improves access to care. The organization in question provides a variety of pediatric therapy services that are highly resource constrained relative to demand. This is a consequence of a multitude of factors including a limited supply of licensed treatment professionals, growing service demand over the past 3 decades, and budget constraints. Furthermore, the organization has reported growing demand for services, particularly SLP, after COVID-19 pandemic restrictions eased. This may be in part due to the reduction in service capacity during the peaks of the pandemic resulting in a greater backlog of patients and is a trend that has been observed in other countries, such as Ireland.^1^ As a result, there is a pressing need to generate treatment capacity by improving system efficiency, particularly as the system is currently overburdened, evidenced by a lengthy and growing waitlist. For example, wait times for SLP assessments are already 4 times the recommended 8-wk maximum,^23^ and these waiting times are endemic to the field.^4^

## Data

### Participants

Anonymized patient and booking data were obtained from a southwestern Ontario nonprofit pediatric rehabilitation organization. Appointment schedules and cancellations, as well as employee-worked and -benefit hours, were recorded in their clinical care database. The organization studied serves multiple patient groups with different needs, ages, and service characteristics. The programs can be divided into 2 primary groups: early years (EY), which refers to programs for patients younger than 3 y, and school aged (SA), which refers to programs for patients aged 5 y and older. Within these groupings, different programs service patients with different needs, namely, SLP, physical therapy (PT), and occupational therapy (OT).

This study focuses on the EY program due to structural differences in the delivery of treatment and scheduling between the 2 programs. Specifically, the EY program serves patients in an outpatient setting, either face to face at one of the organization’s clinics, over the phone, or virtually. The SA program, in contrast, serves patients directly at their schools. Subsequently, cancellations are significantly less common and impactful in the school-based setting, as patients typically cancel only if an emergent reason such as illness prevents them from attending school the day of their appointment.

Focusing on the EY SLP, PT, and OT programs, we have data on 73,482 patient visits, cancellations, and no-shows from June 2021 to October 2023, a basic description of which can be found in Table 1.

**Table 1:** Descriptive Statistics of Full Visit and Cancellation Dataset^a^

Variable	Speech-Language Pathology	Physical Therapy	Occupational Therapy	Total
Visits	48,063	7,179	18,240	73,482
Modality				
Telephone	757 (1.82%)	79 (1.30%)	338 (2.19%)	1,174 (1.60%)
Virtual	10,387 (25.01%)	808 (13.26%)	3,996 (25.89%)	15,191 (20.67%)
In-person	30,384 (73.17%)	5,207 (85.44%)	11,099 (71.92%)	46,690 (63.54%)
Cancellations/no-shows				
Patient cancellation >24 h	2,193 (21.85%)	268 (19.20%)	709 (18.10%)	3,170 (20.65%)
Patient cancellation <24 h	3,124 (31.13%)	548 (39.26%)	1,194 (30.47%)	4,866 (31.70%)
No show	2,035 (20.28%)	200 (14.33%)	962 (24.55%)	3,197 (20.83%)
Clinician cancellation	1,673 (16.67%)	154 (11.03%)	643 (16.41%)	2,470 (16.09%)
COVID-related cancellation	250 (2.49%)	29 (2.08%)	81 (2.07%)	360 (2.35%)
Other nonattendance type	761 (7.58%)	197 (14.10%)	329 (8.40%)	1,287 (8.38%)
Lead time (d)				
Mean	32.69	31.19	34.08	32.89
Median	28.00	27.00	28.00	28.00
Standard deviation	24.21	23.00	25.63	24.47
Maximum	222.00	182.00	222.00	222.00

a Percentages in brackets denote proportions of the total sample size (for each program and overall). Other nonattendance types refer to appointments that did not occur due to weather closures, clients being discharged ahead of a booked appointment, or other external factors.

We make note that between June 2021 and January 2022, nearly all visits were delivered virtually or by telephone due to COVID-related restrictions impacts on service delivery. Furthermore, service was frequently disrupted by pandemic-related events. It is important to take this into account as following the easing of these restrictions entering 2022, service returned to being predominantly delivered in-person, which has a significantly higher association with last-minute cancellation and no-show behavior compared with virtual or telephone appointments. This motivated starting the cancellation rate analysis in January 2022, when, in combination with examining the data and discussions with managers from the organization, it was deemed service went back to a “normal” delivery modality mix and relatively few exogenous disruptions occurred.

### Cancellations

There are various forms of undelivered appointments in the EY program data, including no-shows, patient cancellations, clinician cancellations, COVID-related cancellations, and weather-induced temporary service suspensions. Last-minute cancellations comprise more than 32% of all undelivered appointments, while no-shows account for a further 21% of undelivered appointments, as seen in Table 1. Specific to this organization, historically, 96.9% of cancellations within 24 h of an appointment go unfilled, and 99.3% of no-show appointments go unfilled. Comparatively, 86.3% of appointments with a patient cancellation more than 24 h in advance go unfilled. In the case of an advance cancellation, clinicians may also be able to shift their work hours to book a patient sometime later in the day or week, avoiding an increase in idle time; this reduces the negative impact of advanced cancellations on effective treatment capacity.

**Figure 1:**
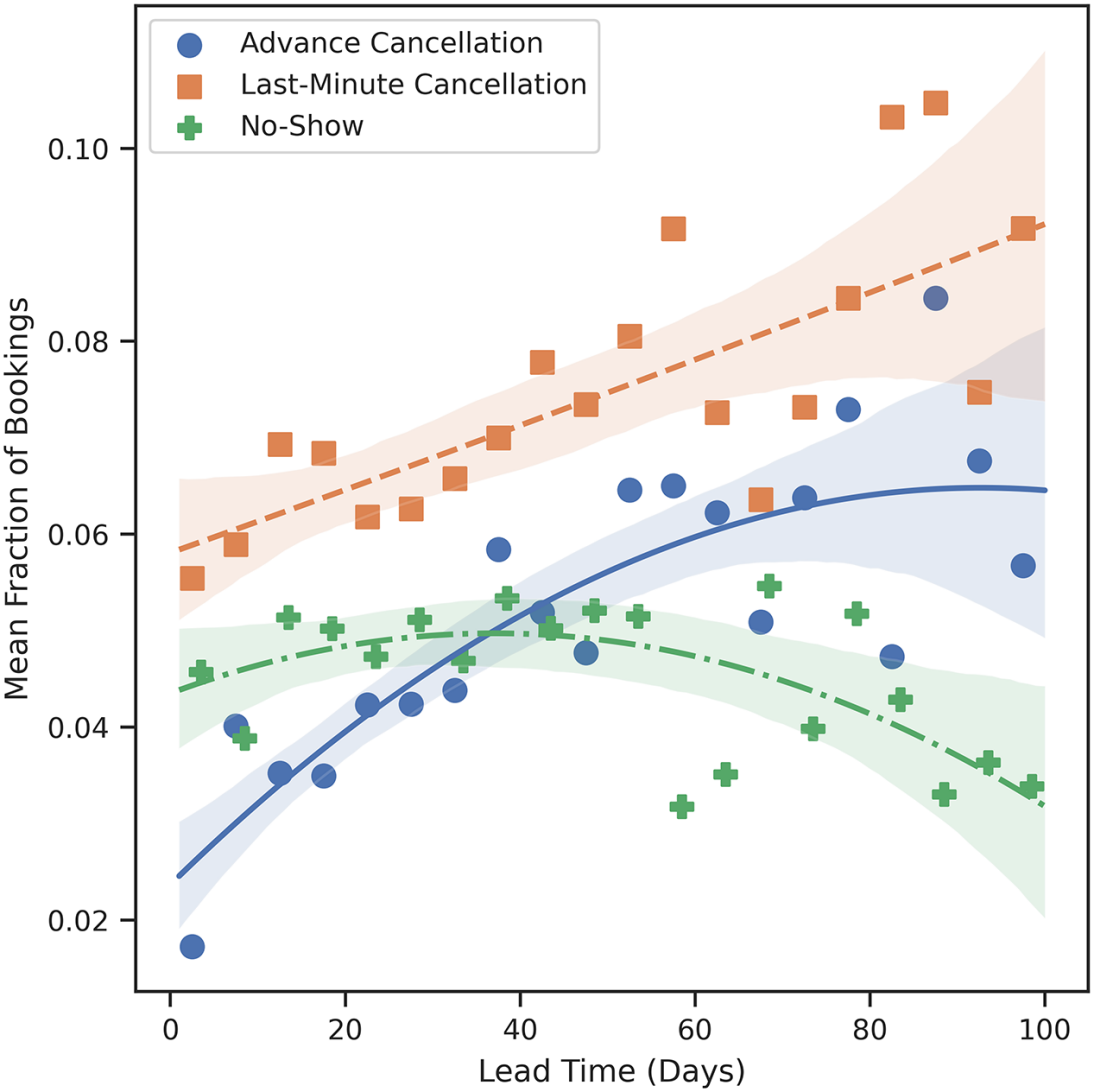
Mean fraction of appointments cancelled or missed as a function of their lead time.

As seen in Figure 1, the average last-minute cancellation rate increases from ∼6% for next-day appointments to ∼9% for appointments booked 100 d in advance. A moderate positive monotonic correlation is found between the lead time and the last-minute cancellation rate (Spearman’s ρ = 0.32, *P* = 0.004). Advanced cancellations also monotonically increase as lead time increases (Spearman’s ρ = 0.72, *P* < 0.001), although the effect diminishes when lead time is large (i.e., lead time >80 d). The rate of no-shows, on the other hand, initially increases as lead times increase. However, as lead time grows beyond ∼40 d, the rate of no-shows decreases. One explanation for this observation is that although the likelihood of a patient not attending their appointment, be it via cancellation or no-show, increases monotonically with lead time, there is an inflection point (∼40 d) when a patient is more likely to provide the clinic with notice of their nonattendance. Thus, although no-show rates decrease as lead times grow beyond 40 d, the overall rate of appointment nonattendance still monotonically increases.

## Policy Change

Motivated primarily by the modeling works of Liu^22^ and Leeftink et al.,^17^ the organization was recommended to reduce its booking horizon from 12 to 4 wk at a policy level. The organization ran a pilot study with 5 clinicians between October 2022 and January 2023; however, there were significant abnormalities in scheduling for several of the included clinicians. Despite these anomalies, the pilot results were promising enough that management moved forward with implementing the policy across the SLP, PT, and OT programs in the EY cohort, beginning in February 2023. Thus, for our analyses, we consider the population of interest to be EY patient appointments in the SLP, PT, and OT programs between January 2022 and October 2023 and the intervention to have occurred in February 2023.

Our primary key performance indicator is the combined last-minute cancellation rate and no-show rate, aggregated weekly. We combine last-minute cancellations and no-shows for multiple reasons. First, cancellations within 24 h of an appointment and no-shows have the same negative impact on the clinic in our setting. As overbooking and walk-in treatment do not occur, last-minute canceled appointment slots are effectively as difficult to fill as no-shows are, leading to equivalent increases in idle time. Second, based on the observed trends with lead time, it is possible that reducing the booking horizon may effectively marginally increase the no-show rate while decreasing the last-minute cancellation rate. As we are primarily interested in the effect of reducing the booking horizon on effective resource usage (i.e., reduced wasted treatment capacity and idle time), we are interested in ascertaining if the overall rate of costly nonattendance (i.e., no-shows plus last-minute cancellations) decreases, increases, or remains the same.

## Methods

### Analysis of Booking Horizon Policy Effects on Lead Time

Before analyzing the trends in cancellation and no-show rates, it is important to ascertain if the policy change influenced lead times. Although some appointments are scheduled by dedicated clinical scheduling assistants, many appointments in the EY programs are scheduled directly by the treating clinician. As such, it is possible that some clinicians did not adhere to the new policy or, due to their panel size, were unable to adhere to the policy at the time it was implemented. The inclusion of data from clinicians whose lead times did not change would likely lead to underidentification of the potential effect of the booking horizon policy change on cancellation and no-show rates. Furthermore, some clinicians left the EY program before the policy change or joined the program after the policy change, complicating the estimation of possible changes in trends associated with the policy change.

To account for these factors, we first subset appointments belonging to clinicians who delivered an average of 5 or more appointments per week before and after the policy change, resulting in a sample of observations from 52 clinicians. This removes observations associated with clinicians who may have been entering or leaving the organization during the period of interest. Given that clinicians perform a significant amount of appointment scheduling themselves, and our lack of an external control group, we want to ensure that the samples in the pre– and post–policy change periods are similar. For example, we do not want to include samples from a clinician who worked only in the pre–policy change period, as we will not observe how the policy change is associated with changes in their scheduling behavior and cancellation/no-show behavior of their clientele. This filtering also removes clinicians who primarily work in other programs within the organization but had picked up the occasional appointment or client within the EY program. As these clinicians work primarily in a different program with different booking policies, in practice they generally follow that program’s policies for all their clients, and thus, their adoption of the reduced booking window policy for the EY program is highly uncertain.

Subsequently, the sample is trimmed to include only clinicians who displayed a statistically significant reduction in appointment lead times after February 2023 (i.e., when the new booking policy was implemented), resulting in a sample of 41 clinicians. Including samples from the 11 removed clinicians would not make sense in this context, as the policy change does not change their scheduling behavior or booking horizon. As with any policy implementation in a large organization, there is a policy-practice gap. Some employees may be resistant to changing their behavior, in this case scheduling behavior, per a new policy until they observe the positive impacts of the policy. Furthermore, clinicians with extremely large panel sizes have reported difficulty managing their panels, something that may have contributed to their lack of adherence to the new policy. Ultimately, the focus of this work is estimating the effect of reducing the booking horizon on cancellation and no-show rates, not examining the barriers to policy adoption. Thus, we leave the question of why the policy did not seem to affect the scheduling patterns of some clinicians as an open research question for future study.

**Figure 2:**
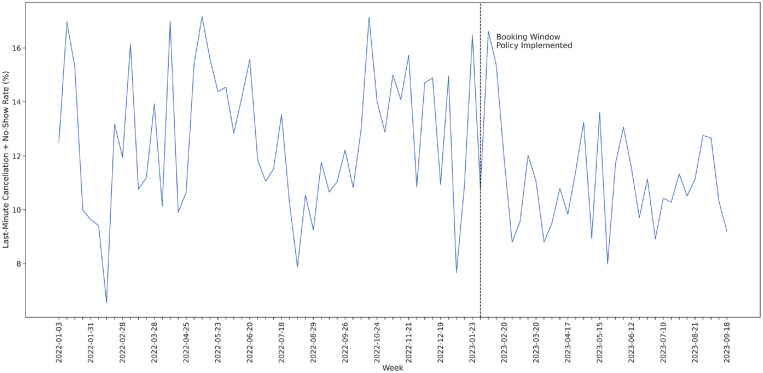
Weekly last-minute cancellation + no-show rate between January 2022 and mid-October 2023.

After performing this data selection, we obtained data on 24,784 appointments delivered by 41 clinicians between January 2022 and October 2023, a description of which can be seen in Table 2. The trend in aggregate weekly last-minute cancellation/no-show rates for this time frame is shown in Figure 2.

**Table 2:** Descriptive Statistics of Visit and Cancellation Data for the *N* = 41 Clinician Cohort Used in the ITS Analysis^a^

Variable	<February 2023	≥February 2023	Total
Visits	13,665	11,119	24,784
Modality			
In-person	13,665	11,119	24,784
Cancellations/no-shows			
Patient cancellation >24 h	701 (20.84%)	519 (22.86%)	1,220 (21.65%)
Patient cancellation <24 h	1,065 (31.66%)	746 (32.86%)	1,811 (32.14%)
No show	677 (20.12%)	484 (21.13%)	1,161 (20.60%)
Clinician cancellation	508 (15.10%)	302 (13.30%)	810 (14.38%)
COVID-related cancellation	184 (5.65%)	53 (2.33%)	237 (4.21%)
Other nonattendance type	196 (5.83%)	166 (7.31%)	395 (7.02%)
Lead time (d)			
Mean	40.22	32.40	36.70
Median	35.00	28.00	31.00
Standard deviation	28.10	23.19	26.30
Maximum	222.00	185.00	222.00

ITS, interrupted time series.

a Percentages in brackets denote proportions of the total sample size (for each period and overall). Other nonattendance types refer to appointments that did not occur due to weather closures, clients being discharged ahead of a booked appointment, or other external factors.

### Interrupted Time-Series Analysis

#### Traditional interrupted time series

Instead of using a simple before-after design to estimate the effect of the policy change, we opted to use an interrupted time-series (ITS) analysis. This allows for the underlying trend in the cancellation rate to be accounted for and reduces the chances of confounding being an issue, as population characteristics vary little over time.^24^ The advantage of ITS over, for example, an ordinary least squares regression, is the ability to use models such as autoregressive (AR), moving average (MA), or AR MA (ARIMA) models to account for autocorrelation in the data. AR models estimate the value of a time series at time *t* as a weighted linear combination of its past values, known as lags. An AR model can be defined as

$$X_{t}=\sum_{i=1}^{p}\beta X_{t-i}+\epsilon_{t}=\sum_{i=1}^{p}AR\left(i\right)X_{t-i}+\epsilon_{t}$$

where *p* denotes the number of lags to include. For the rest of this article, we will denote the coefficients of the AR terms as AR(*i*), where *i* indicates the lag. Similarly, we can define the MA model as follows:

$$X_{t}=\sum_{i=1}^{p}\beta \epsilon_{t-i}+\epsilon_{t}=\sum_{i=1}^{p}MA\left(i\right)\epsilon_{t-i}+\epsilon_{t}$$

An ARIMA model is a combination of the AR and MA models and can be formulated as

$$\begin{array}{l} X_{t}=\sum_{i=1}^{p}\beta X_{t-i}+\sum_{i=1}^{q}\gamma \epsilon_{t-i}+\epsilon_{t}=\sum_{i=1}^{p}AR\left(i\right)X_{t-i}\\ +\sum_{i=1}^{q}MA\left(i\right)\epsilon_{t-i}+\epsilon_{t} \end{array}$$

Various approaches exist for using an ARIMA model to estimate the effect(s) of an intervention on the variable of interest. One approach consists of including exogenous regressors (ARIMAX) to model changes in the time series, such as level shifts (LS), temporary changes (TC), and ramps. For example, an LS that occurs at t = τ can be modeled by adding a binary indicator variable to the model, specified by

(1) $$LS_{t}=\{\begin{array}{cc} 1, & if\;t\geq \tau \\ 0, & otherwise \end{array}$$

The effect of an LS is that there is some positive/negative change in the central tendency of the trend that occurs at and persists after t = T. A TC, on the other hand, models a shift in the time series that decays over time.

Based on visual inspection of the time series in Figure 2, it appears as though there is a negative LS in the time series after the booking horizon policy is implemented. To estimate this LS, we fit an ARIMAX model with an exogenous LS variable beginning in February 2023, following the procedure outlined by Schaffer et al.^25^ As the policy change is a modification of the booking horizon, there is a potential for a lag in effects. For example, if the booking horizon policy changes on February 1, 2023, reducing from 12 wk to 4 wk, there would still be the possibility of appointments in the weeks following February 1 that were booked under the old policy. Motivated by this concept, multiple ARIMAX models are fit with an LS beginning on January 30; February 6, 13, 20, and 27; March 4, 11, and 18; and the intervention date that produces the lowest Akaike information criterion (AIC) is chosen. If for the given intervention date multiple model specifications (i.e., AR(p) and MA(q) orders) produce similarly minimal AIC values, we use visual validation to determine the best-fitting model.

The number of AR and MA terms to include in the model is determined primarily by identifying the combination with the lowest AIC. This choice of AR and MA terms is validated with visual inspection of the time series’ autocorrelation function (ACF) and partial ACF plots (see Figure 4 in Supplementary Appendix A.1).

#### Robust ITS

Aside from hypothesizing that reducing the booking horizon at the policy level will reduce the rate of last-minute cancellations and no-shows, we also hypothesize that it will reduce the variability in these rates, as the range of possible lead times will be condensed. To estimate possible changes in the variability of last-minute cancellation and no-show rates, robust segmented regression modeling is used as it allows for the estimation of changes in the variance of the time series pre- and post-intervention. This work follows the robust-ITS approach presented by Cruz et al.^26^ Their method enables using the data to pick the “best” intervention time point and subsequent estimation of changes in variance if the residuals of the pre- and post-intervention regressions do not exhibit significant autocorrelation. In the case of this study, these conditions hold, and the overall LS and change in slope are validated against the regular ITS approach results as a robustness check.

## Results

Following the methods outlined in the “Traditional ITS” section, it is estimated that the change point occurs on the week of February 27, 2023. The best-fitting model, determined by the lowest AIC score, has 2 AR lags and 2 MA lags, with a single exogenous LS, the results of which are presented in Table 3.

**Table 3:** Parameter Estimates for ARIMAX Model Fitting the Last-Minute Cancellation + No-Show Rate Time Series^a^

Parameter	Estimate
Intercept	1.463 (1.491)
Level shift associated with policy change on February 27, 2023	−1.853*** (0.474)
AR(1)	1.854*** (0.130)
AR(2)	−0.905*** (0.106)
MA(1)	−1.968*** (0.603)
MA(2)	0.970 (0.577)
σ^2^	4.269 (2.802)

a AR(t) and MA(t) indicate, respectively, the autoregressive and moving average terms of the *t*^th^ time lag. Standard errors are in parentheses.

*** *P* ≤ 0.001.

**Figure 3:**
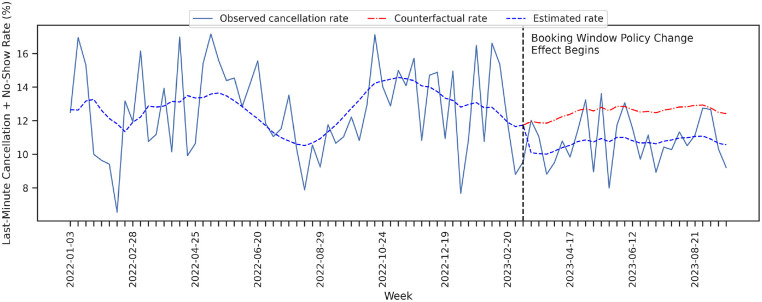
In blue is the time-series model fit that shows the exogenous level shift at the time of the policy change. In red is the time-series model counterfactual estimation, had the policy change not occurred.

The model estimates that there is a statistically and economically significant drop of 1.85% in the mean weekly last-minute cancellation and no-show rate associated with the policy change. This represents a relative reduction of 15.70% compared with the mean prechange point rate. The model fit and counterfactual estimation are presented in Figure 3. The counterfactual rate estimates what the mean weekly last-minute cancellation and no-show rate would have been had the booking horizon policy not been implemented.

Next, the last-minute cancellation and no-show rate was analyzed using the robust-ITS approach, with a focus on estimating the change in variance associated with the intervention. The estimated best-fitting change point was February 13, 2023, and the first-stage regression results can be seen in Table 4. Most importantly, we note that the change in the mean level is −1.02%, indicating that reducing the booking horizon at the policy level did lead to a significant and lasting reduction in the weekly last-minute cancellation and no-show rate. The absolute change in level is equivalent to an 8.07% reduction relative to the preintervention mean weekly last-minute cancellation and no-show rate.

**Table 4:** Parameter Estimates for Robust-ITS Model Fitting the Last-Minute Cancellation + No-Show Rate Time Series^a^

Parameter	Estimate
Intercept pre-change point	12.312*** (0.713)
Intercept post-change point	11.089*** (0.613)
Change in intercepts	−1.224*** (0.940)
Slope pre-change point	0.014 (0.023)
Slope post-change point	−0.012 (0.036)
Change in slope	−0.026*** (0.043)
Change in the mean level (pre- to post-change point)	−1.018***

a Standard errors are in parentheses.

*** *P* ≤ 0.001.

Figure 5 in the supplementary material presents the studentized residuals after modeling the mean. All but 2 residuals reside between the rule of thumb ±2 and do not exhibit any clear patterns, suggesting that robust ITS adequately models the mean last-minute cancellation and no-show rate. Furthermore, we note that the residuals appear to act as white noise according to the ACF plots in Figure 6 (see supplementary material). To confirm the lack of significant correlation, the pre- and post-change point phase residuals are modeled using an AR(1) process separately. The estimates for the AR(1) coefficients can be found in Table 5 and show that there is no significant evidence of autocorrelation in either period. Due to the lack of significant autocorrelation, an *F* test can be used to compare the estimated variances in each period. We find that after the booking horizon is reduced at the policy level, the variance in the weekly last-minute cancellation and no-show rates decreases by ∼48.18%. This further indicates the benefit of the new booking horizon policy as the last-minute cancellation and no-show rate not only decreases significantly but also becomes more stable.

**Table 5:** Estimates for the AR(1) Coefficients Pre- and Post-change Point and the Estimated Increase in the AR(1) Coefficient Post-change Point^a^

Parameter	Estimate
AR(1) coefficient pre-change point	0.174 (0.143)
AR(1) coefficient post-change point	0.206 (0.183)
Difference in AR(1) coefficients	0.032
Variance pre-change point	6.584 (1.613)
Variance post-change point	3.412 (0.926)
Variance difference (post–pre)	−3.172
Variance comparison *F* statistic (*P* value)	1.930 (0.032)

a The estimated variances pre- and post-change point and the *F* statistic and *P* value corresponding to the difference in variances.

## Discussion

The primary result of this study was validation that standardizing and decreasing the booking horizon correlates with a significant reduction in the rate of highly disruptive appointment nonattendances. In our setting, we show that reducing the booking horizon from 12 to 4 wk reduces the weekly combined rate of last-minute cancellations and no-shows by an absolute amount of 1.02% to 1.85% (a relative reduction of 8.075 to 15.70%). These results provide real-world support for modeling studies such as those of Liu^22^ and Leeftink et al.^17^ These studies create mathematical models for optimizing the booking horizon given lead-time–dependent cancellation and no-show rates and provide numerical experiments based on lead-time cancellation/no-show associations reported in other studies. They do not provide, however, experimental or observational results for real-world settings in which the booking horizon was changed. We further demonstrate that reducing the booking horizon is associated with a significant reduction in the variance of weekly last-minute cancellations and no-show rates. In the case of this study, the reduction was 48.18%. This finding is important, as more consistent rates make mitigating idle time increases associated with implementing other strategies, such as overbooking, easier.

It is also important to understand the benefit of this policy in the context of our partner organization and why it may be a worthwhile policy to implement in organizations that face similar resource constraints. Given the large volume of patients that the organization sees, a small but significant drop in the last-minute cancellation and no-show rate translates to a significant increase in the number of patients seen per week. For example, the study cohort clinicians booked an average of 363 appointments per week during the post-change period. Using a counterfactual analysis (i.e., had the booking horizon policy not changed) based on the ITS model, it is estimated that, on average, an extra 6.17 patients were seen during the post-change period (i.e., attended their appointment rather than cancel/no-show). In the case of our partner organization, this is the equivalent of increasing the number of clinicians on staff by 0.4 full-time equivalents. This effectively increases the amount of treatment being delivered to patients without either 1) requiring higher organizational spending to hire more staff or 2) requiring employees to extend their hours, which can contribute to burnout, increase overtime spending, or violate union constraints.

The main limitation of this study is that it is based on observational rather than experimental data. Unfortunately, as the policy was implemented across the full EY program, it is difficult to construct a control group of clinicians whom we can say for certain were not influenced by this policy in some way. While the organization has other programs, such as the SA program, the difference in treatment styles and appointment modalities causes different cancellation behaviors, precluding their use as control groups. Therefore, although we know what the policy change was and when it was introduced, there remains the possibility that there were also unknown and unrelated changes to the EY system over time that affected cancellation and no-show rates. We try to account for this in 2 ways: 1) by isolating our analysis to clinicians (and their clientele) with sufficient longitudinal data volumes pre- and post-policy change and 2) by using 2 forms of ITS analysis to confirm that our results are robust. Future work could go in 2 directions. First, experimental studies could be performed in which patients and clinicians are randomized between treatments (i.e., booking horizon policies), and the resulting cancellation and no-show rates during the same period could be compared. Another opportunity exists to expand the current modeling research about optimizing booking horizons to reduce cancellations and no-shows. For instance, models could be developed that consider organizations’ cancellation time-dependent ability to fill canceled appointment slots, something that the current literature does not account for. This dynamic may change the optimal booking horizon compared with existing booking-horizon optimization models^17,22^ as not all cancellations would incur the same cost (i.e., those canceled further in advance cost less).

## Conclusion

Previous empirical studies have highlighted appointment lead time as being positively correlated with cancellation and no-show probability, and modeling work has shown that optimizing the booking horizon considering cancellation and no-show behavior can decrease the occurrence of these events, in the case of a hard booking horizon maximum. We conducted an observational study to assess the effect of reducing the booking horizon at the policy level in a setting with a soft maximum horizon. The analysis showed that reducing the booking horizon at the policy level is associated with a significant reduction in the level and variance of the combined last-minute cancellation and no-show rate in an outpatient pediatric care setting with significant resource constraints.

## Supplemental Material

sj-docx-1-mpp-10.1177_23814683241298673 – Supplemental material for Effects of Booking Horizon Reduction on Cancellation Rates: An Experimental Analysis in Pediatric Outpatient Care Supplemental material, sj-docx-1-mpp-10.1177_23814683241298673 for Effects of Booking Horizon Reduction on Cancellation Rates: An Experimental Analysis in Pediatric Outpatient Care by Benjamin Ravenscroft, Hossein Abouee Mehrizi and Brendan Wylie-Toal in MDM Policy & Practice
