# Supplementary material for: Effects of Booking Horizon Reduction on Cancellation Rates: An Experimental Analysis in Pediatric Outpatient Care
Source: MDM Policy Pract. 2024 Nov 18;9(2):23814683241298673. doi: 10.1177/23814683241298673 (PMC11574887; doi:10.1177/23814683241298673)
Supplement: sj-docx-1-mpp-10.1177_23814683241298673 – Supplemental material for Effects of Booking Horizon Reduction on Cancellation Rates: An Experimental Analysis in Pediatric Outpatient Care [file sj-docx-1-mpp-10.1177_23814683241298673.docx]

# Appendix A.1: Supplementary Figures


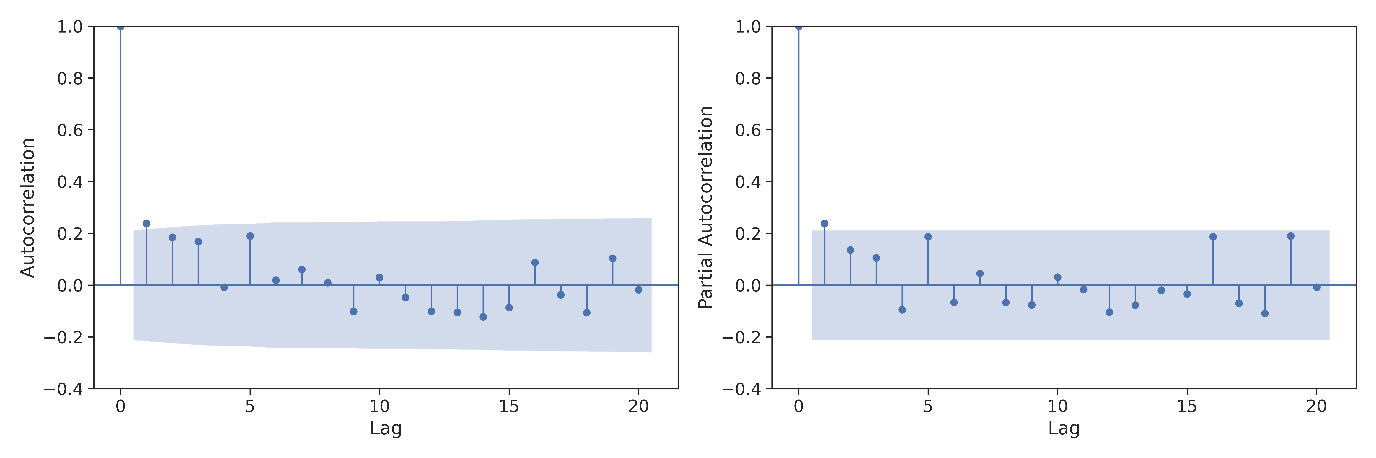


Figure 4: ACF and PACF plots for the last-minute cancellation + no-show rate time series.


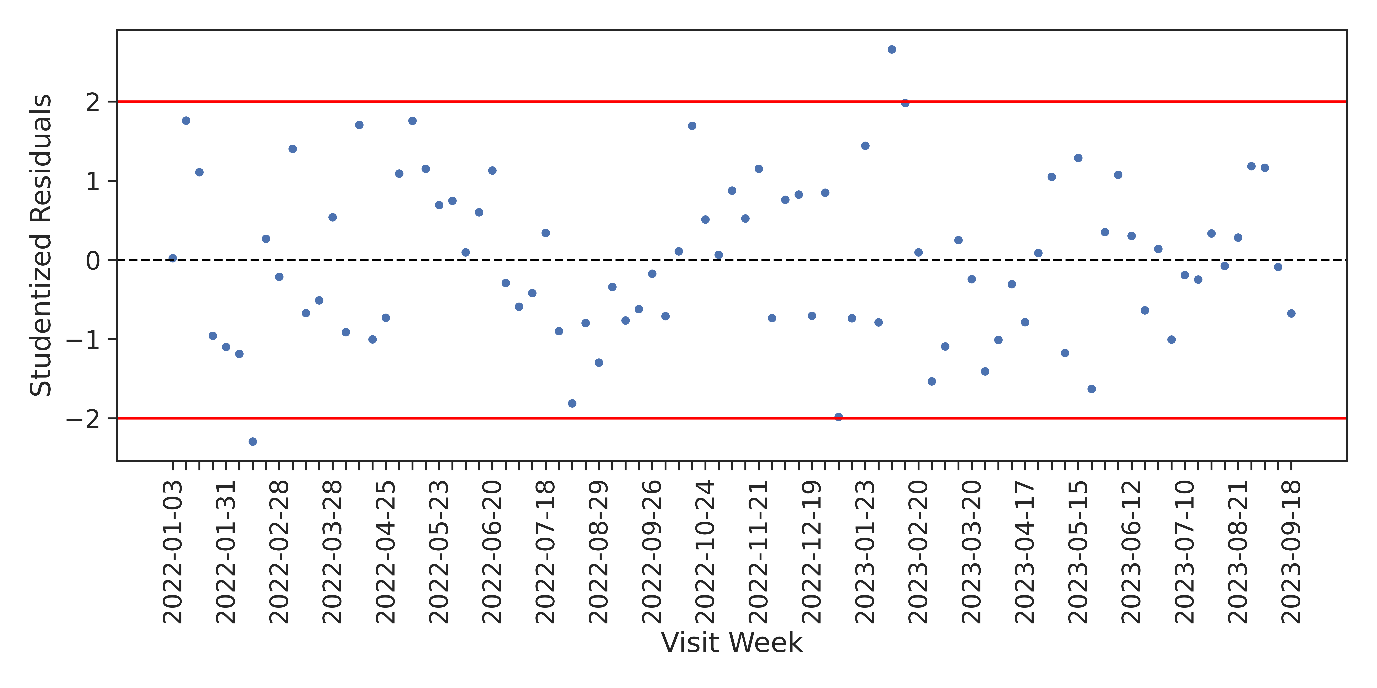


Figure 5: The studentized residuals of the robust-ITS estimated last-minute cancellation and no-show rate mean. The studentized residuals do not show any clear patterns and are closely centered around zero. The rule of thumb, 2, is marked in red.


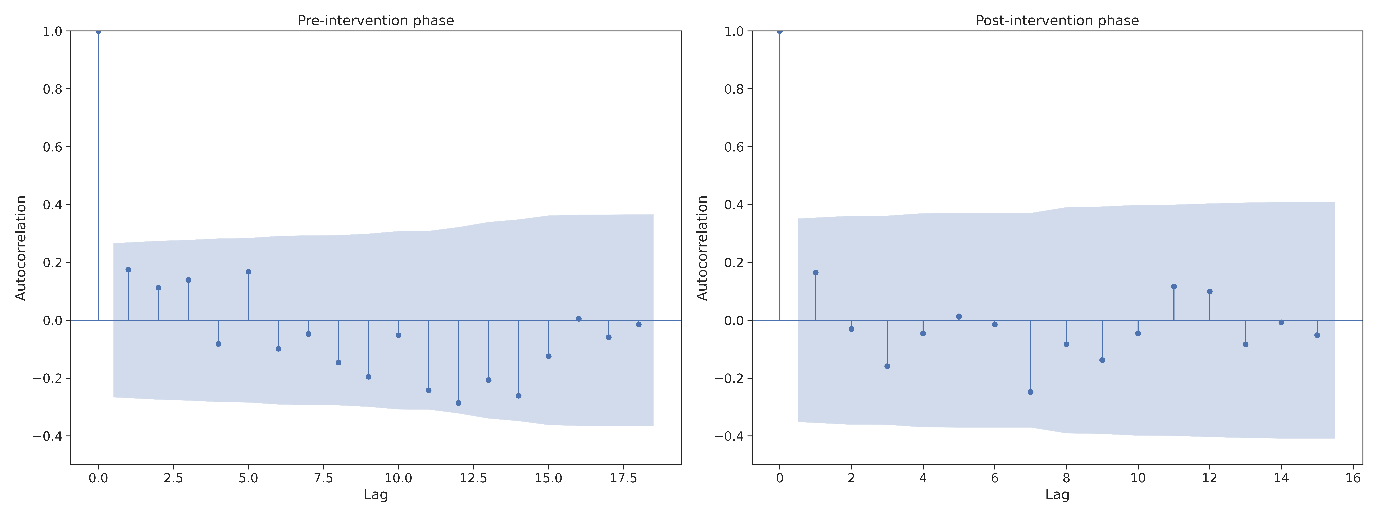


Figure 6: Autocorrelation function (ACF) plots of the robust-ITS estimated last-minute cancellation and no-show rate mean. The autocorrelation is small and decreasing at lags greater than zero, indicating the residuals behave as white noise.


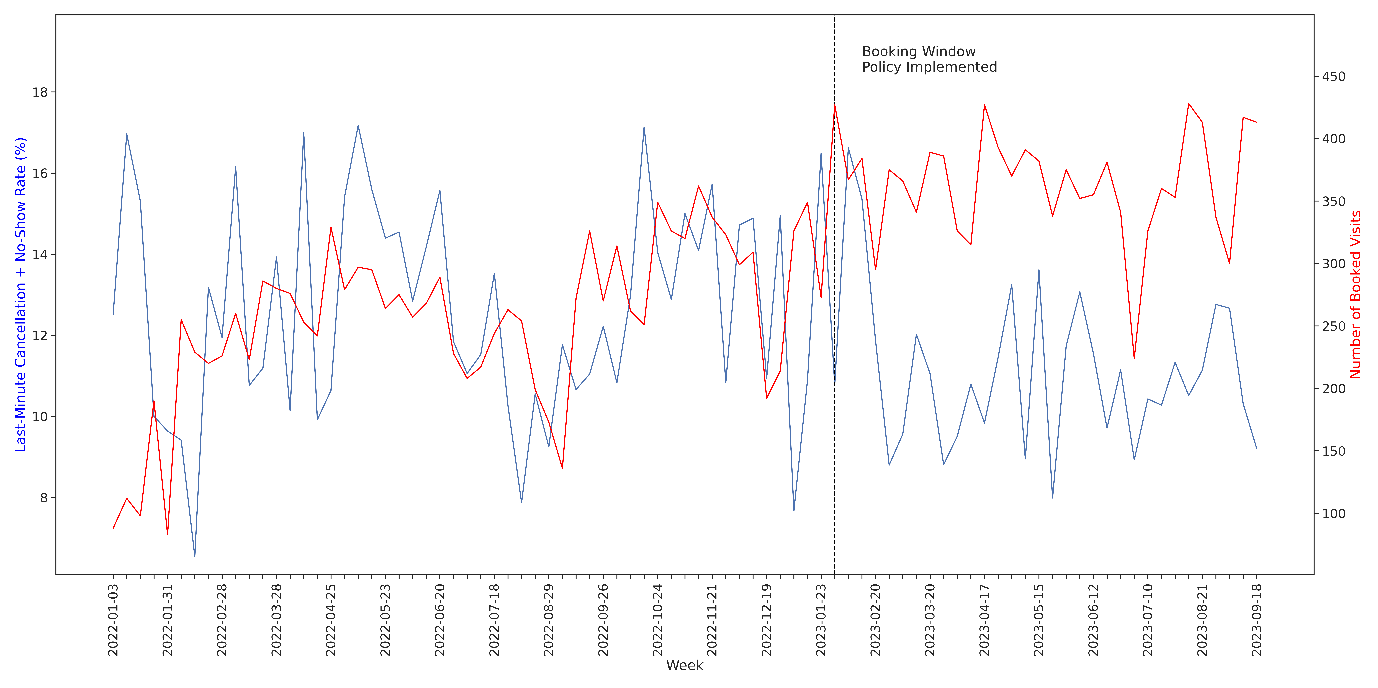


Figure 7: Weekly last-minute cancellation + no-show rate and number of booked visits between January 2022 and mid-October 2023.
